# Correction to “Presence of exacerbating factors of persistent perceptual‐postural dizziness in patients with vestibular symptoms at initial presentation”

**DOI:** 10.1002/lio2.1179

**Published:** 2023-11-30

Kabaya K , Tamai H , Okajima A , Minakata T , Kondo M , Nakayama M , Iwasaki S . Presence of exacerbating factors of persistent perceptual‐postural dizziness in patients with vestibular symptoms at initial presentation. Laryngoscope Investig Otolaryngol 2022 Jan 25;7(2):499–505. doi: 10.1002/lio2.735.PMC900815635434346

The identified calculation error is being corrected in two places as noted below.

On the third line in the Results section of the Abstract, the percentage after “eight patients” should be “(5.2%)” instead of “(10.3%)” as follows:

According to the follow up for an average of 543.3 days after the initial presentation, eight patients (5.2%) developed PPPD.

In the 4th paragraph of the “Results” section, the percentage after “eight patients” should be “(5.2%)” instead of “(10.3%)” as follows:

Patients were followed up for an average of 543.3 ± 203.2 days (range 243–945 days) from the initial presentation and during this time, eight patients (5.2%) developed PPPD (Table 3).

We apologize for this error.

